# I. Ante-partum Vaginal Prolapse. II. Excessive Œdema of the Head

**Published:** 1898-02

**Authors:** W. H. Dalrymple

**Affiliations:** Baton Rouge, La.


### I. ANTE-PARTUM VAGINAL PROLAPSE.

### II. EXCESSIVE ŒDEMA OF THE HEAD.

By W. H. Dalrymple, M.R.C.V.S.,
BATON ROUGE, LA.

I. A few weeks ago I was requested to examine a cow in a condition with which the owner was quite unfamiliar—"a protrusion from behind which might require amputation." I found a prolapsed vagina of at least six days' standing. The exact history of the case is uncertain, but the following is as much of it as I could ascertain. The animal was supposed to be pregnant, and was turned out on to a common pasture along with other cattle, and, I believe, other animals, as horses and mules. Six days previous to my seeing her she was noticed by a colored boy to be in the condition mentioned, but the lad never thought of notifying anyone of what he had seen, consequently the cow was allowed to remain as she was until the day I was called in to see her. The condition of the prolapsed organ can well be imagined; tumefied, callous, with strangulated bloodvessels, etc.

My first thought was the reduction of the prolapse, and to accomplish which I had the animal brought to the owner's stable in town, a distance of about a mile. After cleansing the parts thoroughly with a warm creolin solution, and well lubricating, I was successful, but not before exercising a good deal of patience and expending a considerable amount of physical strength. A web-truss was then applied, and no further inversion took place. The cause of the accident was surmised to be abortion, resulting from an injury while at pasture. Everything went on all right until about a week afterward, when the cow gave birth to a dead fœtus in a state of decomposition. I did not suspect a fœtus *in utero*, as the external appearance of the mother was not such as to attract my attention, and, after placing the vagina in situ, the os was so callous and contracted that I was unable to introduce more than two fingers, so that exploration of the uterus was impossible, otherwise I would no doubt have discovered the fœtus. The tissues of the parts having largely resumed a normal condition, no doubt permitted of relaxation, and the passage outward of the fœtus. The animal is apparently doing well.

Ante-partum prolapse being much less frequent than after parturition, is my excuse for recording this case.

II. Two weeks ago a bay gelding was brought to my infirmary with his head enormously enlarged, so much so, in fact, that it was held so low, on account of its weight, as to almost touch the ground. The whole of the soft tissues seemed to be engorged, giving one the idea that if the skin of the face was merely touched with the point of knife or lancet, it would burst open. Respiration was very much obstructed, the nasal cavities being apparently occluded. The tongue was very much swollen, and almost immovable. In fact, the head was of such a size that it would have been impossible for the animal to have got it into an ordinary sized bucket. The tumefaction was confined entirely to the tissues of the head those of the neck remaining normal. On the following morning the condition was, if anything, worse, due possibly to the horse carrying his head in a dependent position during the previous night. As death from asphyxia seemed imminent, I performed tracheotomy, after which the patient seemed to completely collapse, remaining recumbent for two hours. He revived, however, and shortly afterward partook of, with great difficulty, some very fluid gruel, placed in a deep tub, so that he could immerse his mouth sufficiently to suck a little. From that time the œdema seemed gradually to disappear.

While convalescing from the above condition the animal seemed to be affected with incipient diabetes; but whether this was merely incidental, or was dependent upon the former condition, I am not prepared to say. I treated the polyuria with iodide of potash, and fluid-extract of gentian, and the animal was discharged on the twelfth day. Histories of cases are among the rarest things to be got hold of in this part of the country, and this one proves no exception to the rule. The œdema seemed to be altogether local, and was only observed the evening before the animal was brought to me, but as to predisposing causes, it is only a matter of conjecture. The horse could not have had any debilitating disease previously, as he was at work up to the time the head was noticed enlarged.

Möller, in his work on surgery (Dollar's translation, p. 57), records a condition which seems very similar to the one under consideration, but I was unable to find any traces whatever of injury, and I am sorry to say that I did not make an examination of serum from the head. The illustration given on the page above mentioned is a fair representation of my case, but the deformity is not nearly so great as in the case I treated.

